# Two gonad-infecting species of *Philometra* (Nematoda: Philometridae) from groupers (Serranidae) off Tunisia, with a key to *Philometra* species infecting serranid gonads

**DOI:** 10.1051/parasite/2016008

**Published:** 2016-03-08

**Authors:** František Moravec, Amira Chaabane, Jean-Lou Justine, Lassad Neifar

**Affiliations:** 1 Institute of Parasitology, Biology Centre of the Czech Academy of Sciences Branišovská 31 370 05 České Budějovice Czech Republic; 2 Laboratoire de Biodiversité et Écosystèmes Aquatiques, Faculté des Sciences de Sfax (FSS), Université de Sfax BP 1171 3038 Sfax Tunisia; 3 ISYEB, Institut Systématique, Évolution, Biodiversité, UMR7205 CNRS, EPHE, MNHN, UPMC, Muséum National d’Histoire Naturelle, Sorbonne Universités CP51, 55 Rue Buffon 75231 Paris cedex 05 France

**Keywords:** Philometridae, Fish ovary, *Philometra inexpectata* n. sp., *Philometra jordanoi*, Tunisia, Groupers, Mediterranean Sea

## Abstract

Based on light and scanning electron microscopical studies of nematode specimens (males and mature females) collected from the ovary of groupers (Serranidae, Perciformes) in the Mediterranean Sea off Tunisia (near Tunis and Sfax), two gonad-infecting species of *Philometra* Costa, 1845 (Nematoda, Philometridae) are reported: *Philometra inexpectata* n. sp. from the mottled grouper *Mycteroperca rubra* and *P*. *jordanoi* (López-Neyra, 1951) from the dusky grouper *Epinephelus marginatus*. Identification of both fish species was confirmed by molecular barcoding. The new species is mainly characterized by the length of equally long spicules (147–165 μm), the gubernaculum (63–93 μm long) bearing at the tip two dorsolateral lamellar parts separated from each other by a smooth median field, a V-shaped mound on the male caudal extremity, the presence of a pair of large caudal papillae located posterior to the cloaca and by the body length of the males (1.97–2.43 mm). *Philometra inexpectata* n. sp. is the fifth known gonad-infecting philometrid species parasitizing serranid fishes in the Mediterranean region. The males of *P*. *jordanoi* were examined by scanning electron microscopy for the first time; this detailed study revealed some new taxonomically important morphological features, such as the number and arrangement of cephalic and caudal papillae, presence of amphids and phasmids and mainly the lamellate structures at the posterior end of the gubernaculum. A key to gonad-infecting species of *Philometra* parasitic in serranid fishes is provided.

## Abstract (fr)

Sur la base d’études, au microscope photonique et électronique, de spécimens de nématodes (mâles et femelles mûres) recueillis dans les ovaires de mérous (Serranidae, Perciformes) de la mer Méditerranée au large de la Tunisie (près de Tunis et Sfax), deux espèces de *Philometra* Costa, 1845 (Nematoda, Philometridae) parasites de gonades sont signalées : *Philometra inexpectata* n. sp. du mérou royal *Mycteroperca rubra* et *P. jordanoi* (López-Neyra, 1951) du mérou noir *Epinephelus marginatus*. L’identification des deux espèces de poissons a été confirmée par les codes-barres moléculaires. La nouvelle espèce est caractérisée principalement par la longueur des spicules (147-165 μm), de tailles égales, le gubernaculum (63-93 μm de long) portant à sa pointe deux parties lamellaires dorsolatérales séparées l’une de l’autre par un champ médian lisse, une bosse en forme de V sur l’extrémité caudale du mâle, la présence d’une paire de grandes papilles caudales situées en arrière du cloaque et par la longueur du corps des mâles (1.97-2.43 mm). *Philometra inexpectata* n. sp. est la cinquième espèce de Philometridae des gonades de poissons Serranidae dans la région méditerranéenne. Les mâles de *P. jordanoi* ont été examinés par microscopie électronique à balayage pour la première fois ; cette étude détaillée a révélé quelques nouvelles caractéristiques morphologiques taxinomiquement importantes, telles que le nombre et la disposition des papilles céphaliques et caudales, la présence d’amphides et de phasmides et principalement les structures lamellaires à l’extrémité postérieure du gubernaculum. Une clé des espèces de *Philometra* parasites des poissons Serranidae est fournie.

## Introduction

Gonad-infecting species of philometrid nematodes (Philometridae) are widely distributed in marine fishes of the Atlantic, Indian and Pacific Oceans, and sometimes occur in brackish-water environments [21, 26]. These parasites may be severely pathogenic in fish ovaries and can affect reproduction [15].

The species identification of these parasites, previously mostly based on the morphology of large-sized females, was rather problematic. However, scanning electron microscopical (SEM) examinations of minute philometrid males made the identification more reliable and indicated considerable species diversity in these nematodes. To date, many gonad-infecting species of *Philometra* Costa, 1845 have been described from a variety of marine fishes belonging to different families and their number is quickly increasing [13, 14, 16, 17, 19, 20, 22, 23, 27–29].

During recent helminthological investigations of some marine fishes in the Mediterranean Sea off the Tunisian coast near Tunis and Sfax [2, 28], males and mature females of philometrid nematodes were collected from the ovary of two species of serranid fishes, the mottled grouper *Mycteroperca rubra* (Bloch) and the dusky grouper *Epinephelus marginatus* (Lowe) (both Serranidae, Perciformes). A close examination revealed that they represent one new and one known insufficiently studied species. Both host species are subtropical marine fishes, which are distributed in the Mediterranean Sea and the eastern Atlantic (*M*. *rubra*) or in the eastern and southern Atlantic and western Indian Oceans (*E*. *marginatus*) and are targeted by commercial and recreational fisheries [3].

## Materials and methods

### Fish and their identification

Fish were purchased at the fish market in Tunis and Sfax, Tunisia; these were previously caught by fishermen in the nearby coastal waters of the Mediterranean Sea. Fish DNA was extracted from tissue samples using the NucleoSpin 96 tissue kit (Macherey-Nagel, Düren, Germany) following the manufacturer’s instructions. Sequences were obtained by amplification and sequencing of a region of the cytochrome oxidase subunit I (COI) mitochondrial gene using the primers FishF1 (5′-TCAACYAATCAYAAAATYGGCAC-3′) and FishR1 (5′-TGATTYTTYGGYCACCCRGAAGT-3′) [34]. Standard PCRs were carried out in 20 μL total volume, containing about 30 ng of DNA, 1 × 10× PCR buffer, 2 mM MgCl_2_, 200 μM mix dNTPs, 150 nM of each primer and 1 unit of Taq polymerase (Qiagen, Hilden, Germany). After an initial denaturation of 3 min at 95 °C, the mitochondrial DNA was amplified through 39 cycles of 15 s at 95 °C, 20 s at 48 °C and 40 s at 72 °C, with a terminal elongation for 5 min at 72 °C. PCR products were purified and sequenced in both directions on 3730xl DNA Analyser 96-capillary sequencer (Applied Biosystems, Waltham, MA, USA). Sequences were edited using CodonCode Aligner software (CodonCode Corporation, Dedham, MA, USA), compared with the GenBank database content using BLAST, and deposited in GenBank under Accession Numbers KU739518–KU739521. Species identification was confirmed using the BOLD identification engine [32]. Since BOLD does not include all sequences available in GenBank but includes others, comments are added for similarities with other sequences. The fish nomenclature adopted follows FishBase [3].

### Nematodes

Philometrid specimens were collected from frozen-thawed fish gonads under the dissecting microscope. They were fixed in hot 70% ethanol and cleared with glycerine for light microscopical (LM) examination. Drawings were made with the aid of a Zeiss drawing attachment. Specimens used for scanning electron microscopy were postfixed in 1% osmium tetroxide (in phosphate buffer), dehydrated through a graded acetone series, critical-point-dried and sputter-coated with gold; they were examined using a JEOL JSM-7401F scanning electron microscope at an accelerating voltage of 4 kV (GB low mode). All measurements are in micrometres unless otherwise indicated.

## Results and discussion

### *Philometra inexpectata* n. sp. (Figs. 1, 2)

urn:lsid:zoobank.org:act:FF5B31D3-A815-42FB-B64A-FC445FF77824

**Figure 1.**
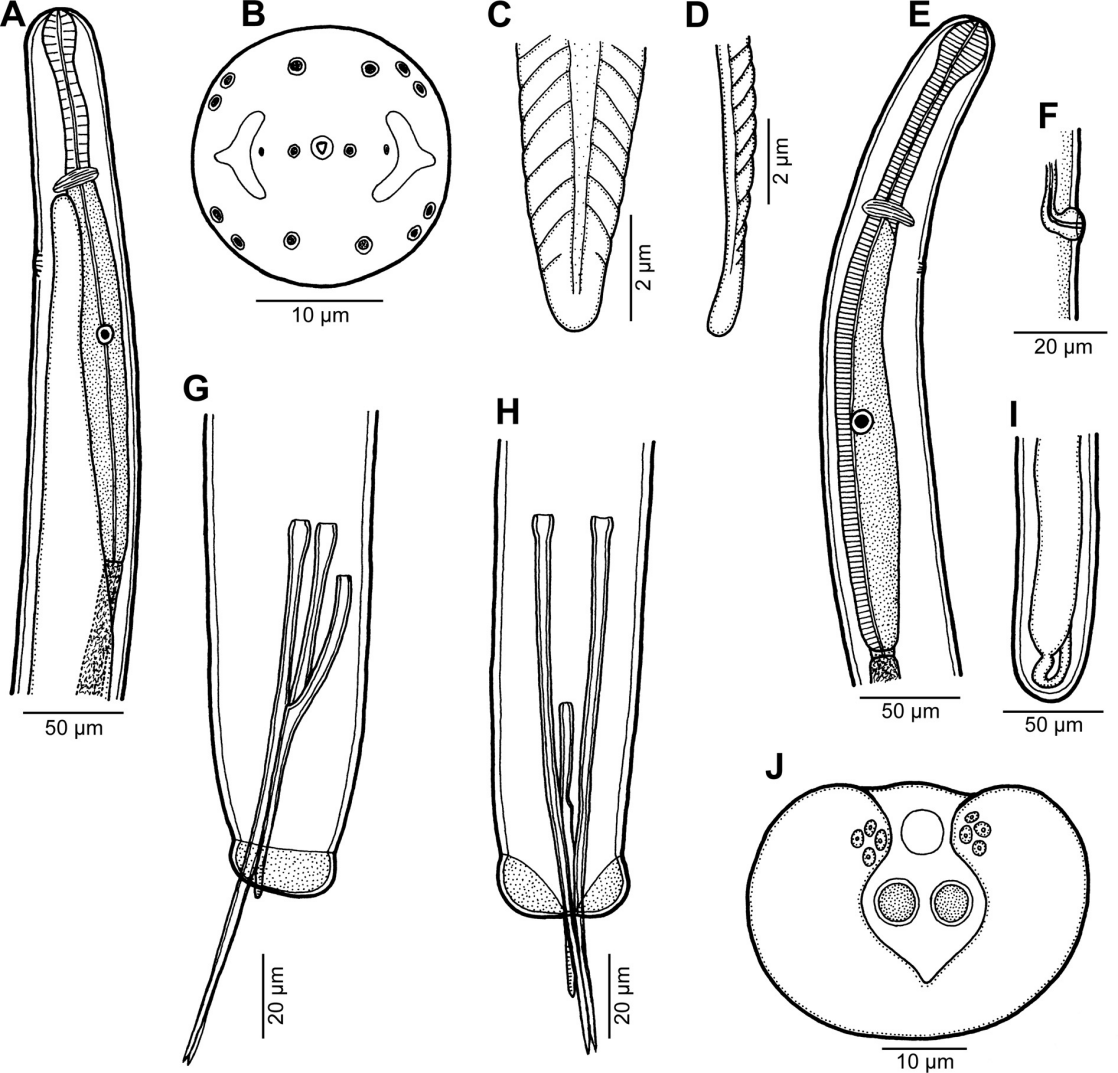
*Philometra inexpectata* n. sp. A: Anterior end of male, lateral view. B: Cephalic end of male, apical view. C, D: Distal end of gubernaculum, dorsal and lateral views, respectively. E: Anterior end of mature female, lateral view. F: Vulva of mature female, lateral view. G, H: Posterior end of male, lateral and ventral views, respectively. I: Posterior end of mature female, lateral view. J: Caudal end of male, apical view.

**Figure 2.**
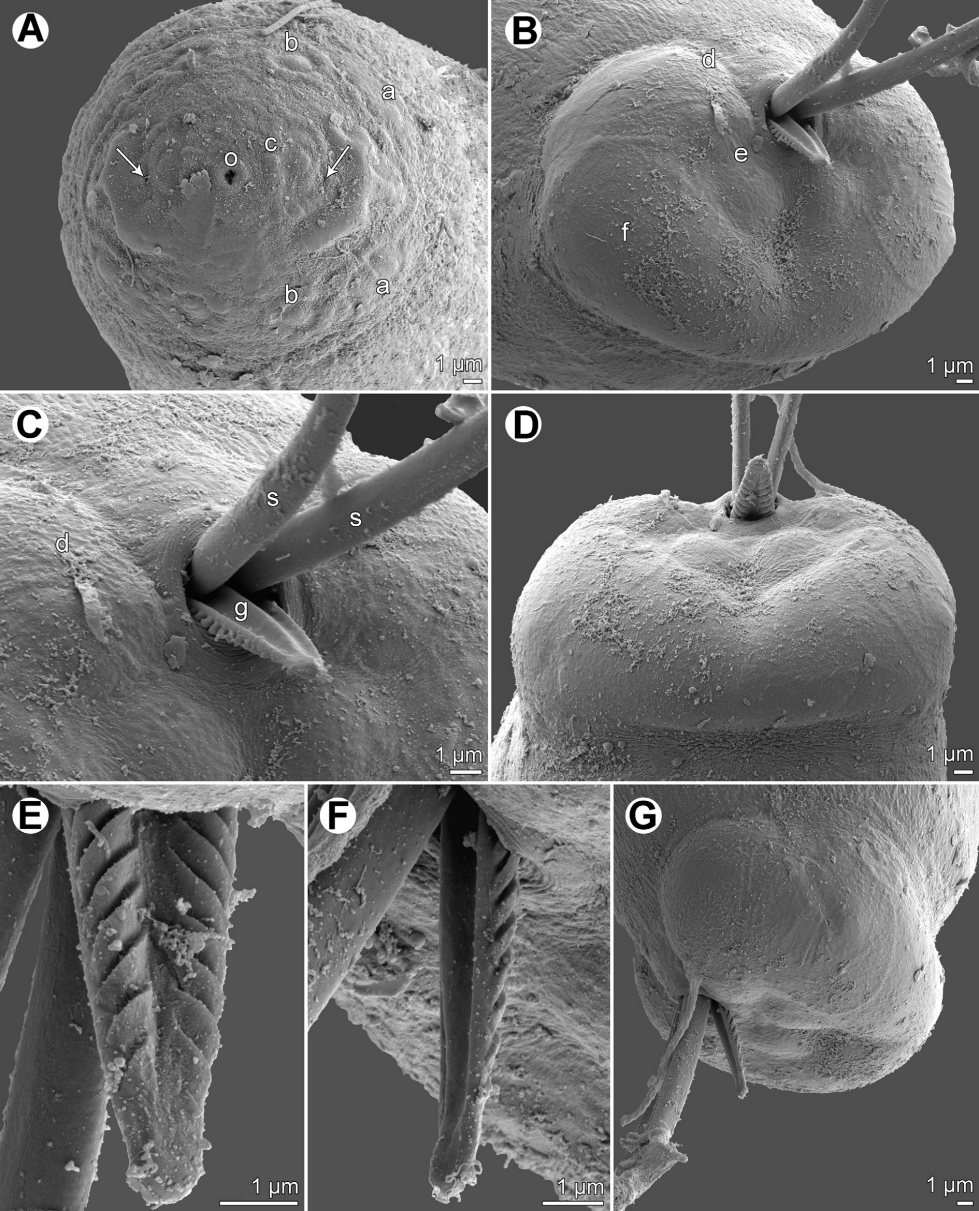
*Philometra inexpectata* n. sp., scanning electron micrographs of male. A: Cephalic end, apical view (arrows indicate amphids). B: Caudal end, apical view. C: Region of cloaca, ventral view. D: Caudal end, dorsal view. E, F: Distal end of gubernaculum, dorsal and lateral views, respectively. G: Caudal end, lateral view. *Abbreviations*: a, submedian pair of external cephalic papillae; b, submedian cephalic papilla of internal circle; c, lateral cephalic papilla of internal circle; d, group of four adanal caudal papillae; e, large papilla posterior to cloaca; f, caudal mound; g, gubernaculum; o, oral aperture; s, spicule.

Type-host: Mottled grouper, *Mycteroperca rubra* (Bloch) (Serranidae, Perciformes). After identifying the fish based upon morphological characteristics, identification was confirmed via barcoding. The COI sequence obtained for our specimen (GenBank Accession Number KU739518) was identical (100% similarity, 94% cover) to a sequence identified as *M. rubra* from off Israel (KF564307; unpublished). BOLD provided similar results. *Mycteroperca rubra* could be confused with the Island grouper *M. fusca* (Lowe), a species from the eastern Atlantic Ocean. Heemstra et al. (2010) [5] reported the presence of *M. fusca* for the first time in the Mediterranean Sea, off Israel. They wrote: “A reasonable possibility is that *M. fusca* entered the Mediterranean through the Strait of Gibraltar, as many Atlantic species do, then expanded its distribution along the North African coast and was overlooked or confused with *M. rubra*.” Unfortunately, no COI sequence of *M. fusca* is available for comparison. Incidentally, we also noted that the sequence we obtained from our specimen was very close (99% similarity, 94% cover) to several other sequences labelled as *M. acutirostris* from Brazil (e.g., KF836485; unpublished); *M. acutirostris* also resembles *M. rubra* and *M. fusca* morphologically, but it has never been reported in the Mediterranean Sea [5]. We thus conclude that the species studied herein from Tunisia is *M. rubra*, but we note that barcoding of more species is needed.

Site of infection: Ovary.

Type-locality: Tunis (fish market), Tunisia (collected 10 September 2015).

Prevalence and intensity: 1 fish infected/1 fish examined; 16 philometrid specimens.

Type-specimens: Holotype, allotype and 39 paratypes, Muséum National d’Histoire Naturelle, Paris, MNHN HEL553-554; 3 male paratypes in the Helminthological Collection of the Institute of Parasitology, Biology Centre of the Czech Academy of Sciences, České Budějovice (Cat. No. N–1109).

Etymology: The specific name *inexpectata* (= unexpected) is a Latin adjective and relates to the fact that gonad-infecting philometrids from *M*. *rubra* were not previously considered to represent a new species.

#### Description

*Male* (13 specimens; measurements of holotype in parentheses): Body filiform, whitish, 1.97–2.43 (2.43) mm long, maximum width at middle of body 54–69 (57); anterior part of body not narrowed just posterior to cephalic end (Fig. 1A). Maximum width/body length ratio 1:33–43 (1:43). Cuticle smooth. Cephalic end rounded, 27–36 (27) wide. Oral aperture small, triangular, surrounded by small circular elevation. Fourteen minute cephalic papillae arranged in 2 circles present: external circle formed by 4 submedian pairs of papillae; internal circle by 4 submedian and 2 lateral papillae. Small lateral amphids just posterior to lateral papillae of internal circle, followed by fairly large lateral crescent-shaped formations of slightly elevated cuticle (Figs. 1B, 2A). Oesophagus 246–360 (249) long, comprising 10–17% (10%) of body length, with inflation at anterior end measuring 27–33 × 18–24 (27 × 18); posterior part of muscular oesophagus overlapped by well-developed oesophageal gland with large cell nucleus; maximum width of gland 15–21 (18). Nerve ring and oesophageal nucleus 105–135 (120) and 171–225 (171) from anterior extremity, respectively. Excretory pore 135–177 (165) from anterior end. Testis extending anteriorly to level of nerve ring (Fig. 1A), usually overlapping posterior portion of oesophagus. Posterior end of body blunt, 27–33 (30) wide, provided with broad V-shaped mound situated laterally and dorsally to cloacal opening. Four adanal pairs of very flat, hardly visible caudal papillae present on anterior parts of caudal mound; additional pair of large subdorsal papillae situated posterior to cloacal aperture (Figs. 1J, 2B–D, G). Phasmids not observed. Spicules slender, needle-like, equally long, with somewhat expanded proximal and sharply pointed distal tips (Figs. 1G, H, 2B–G); length of spicules 147–165 (153), representing 6–8% (7%) of body length. Gubernaculum 63–93 (72) long, with anterior portion somewhat dorsally bent; length of anterior bent part 27–33 (33), representing 31–50% (46%) of entire gubernaculum length (Figs. 1G, H); distal end of gubernaculum with numerous dorsal transverse lamella-like structures demarcating depressed smooth field between them and with two ventral longitudinal grooves (Figs. 1C, D, 2B–G). Length ratio of gubernaculum and spicules 1:1.72–2.38 (1:2.13). Spicules and gubernaculum well sclerotized, yellowish, anterior part of gubernaculum colourless.

*Nongravid female* (3 mature specimens; measurements of allotype in parentheses): Length of body 1.65–1.73 (1.73) mm, maximum width 45–57 (57); maximum width/body length ratio 1:30-38 (1:30). Width of anterior end 30–33 (33). Cephalic structures not studied. Entire oesophagus 270–459 (310) long and 30 (30) wide. Anterior oesophageal bulb 30–36 (30) long, 21–34 (24) wide. Nerve ring and oesophageal nucleus 99–129 (99) and 216–300 (219), respectively, from anterior extremity (Fig. 1E). Vulva and incompletely developed vagina present (Fig. 1F); former situated 1.13–1.21 (1.21) mm from anterior extremity, at 68–70% (70%) of body length. Uterus empty. Posterior end rounded, without caudal projections (Fig. 1I).

#### Remarks

In view of a high degree of host specificity in gonad-infecting species of *Philometra* [22, 23, 27, 28], *P*. *inexpectata* n. sp. is compared with 14 other gonad-infecting nominal species of this genus described from fishes of the perciform family Serranidae: *P*. *aenei* Moravec, Chaabane, Neifar, Gey & Justine, 2016; *P*. *cephalopholidis* Moravec & Justine, 2015; *P*. *charlestonensis* Moravec, de Buron, Baker & González-Solís, 2008; *P*. *cyanopodi* Moravec & Justine, 2008; *P*. *fasciati* Moravec & Justine, 2008; *P*. *hyporthodi* Moravec & Bakenhaster, 2013; *P*. *indica* Moravec & Manoharan, 2014; *P*. *jordanoi* (López-Neyra, 1951); *P*. *margolisi* Moravec, Vidal-Martínez & Aguirre-Macedo, 1995; *P*. *mexicana* Moravec & Salgado-Maldonado, 2007; *P*. *piscaria* Moravec & Justine, 2014; *P*. *serranellicabrillae* Janiszewska, 1949; *P*. *tropica* Moravec & Manoharan, 2014; and *P*. *tunisiensis* Moravec, Chaabane, Neifar, Gey & Justine, 2016. Four of them, *P*. *aenei*, *P*. *jordanoi*, *P*. *serranellicabrillae* and *P*. *tunisiensis*, occur in the Mediterranean region [6, 11, 28].

Morphological and biometrical differences of *P*. *inexpectata* n. sp. from all the above-mentioned species are apparent from the identification key at the end of this paper. To date, only two gonad-infecting species of *Philometra* have been reported to parasitize hosts of the genus *Mycteroperca* Gill: *P*. *charlestonensis*, a parasite of *M*. *phenax* in the North American Atlantic region (USA) [25], and *P*. *lateolabracis* (Yamaguti, 1935) from *M*. *rubra* in the Mediterranean Sea off Turkey (Iskenderun Bay) [18]. However, with respect to the redescription of *P*. *lateolabracis* by Quiazon et al. [30], the nematodes from *M*. *rubra* off Turkey, studied only by LM, were evidently misidentified [12]. Since their morphology and measurements agree with those of the newly described species and because the host species (*M*. *rubra*), localization in the host and the geographical region (Mediterranean Sea) are identical, they evidently belong to *P*. *inexpectata* n. sp.

*Philometra charlestonensis* distinctly differs from *P*. *inexpectata* n. sp. in having shorter spicules (123–141 μm vs. 147–165 μm) and mainly in the male caudal mound consisting of two lateral parts widely separated from each other dorsally (vs. caudal mound V-shaped, not interrupted dorsally).

### *Philometra jordanoi* (López-Neyra, 1951) Yamaguti, 1961 (Figs. 3, 4)

Syn.: *Sanguinofilaria jordanoi* López-Neyra, 1951.

**Figure 3.**
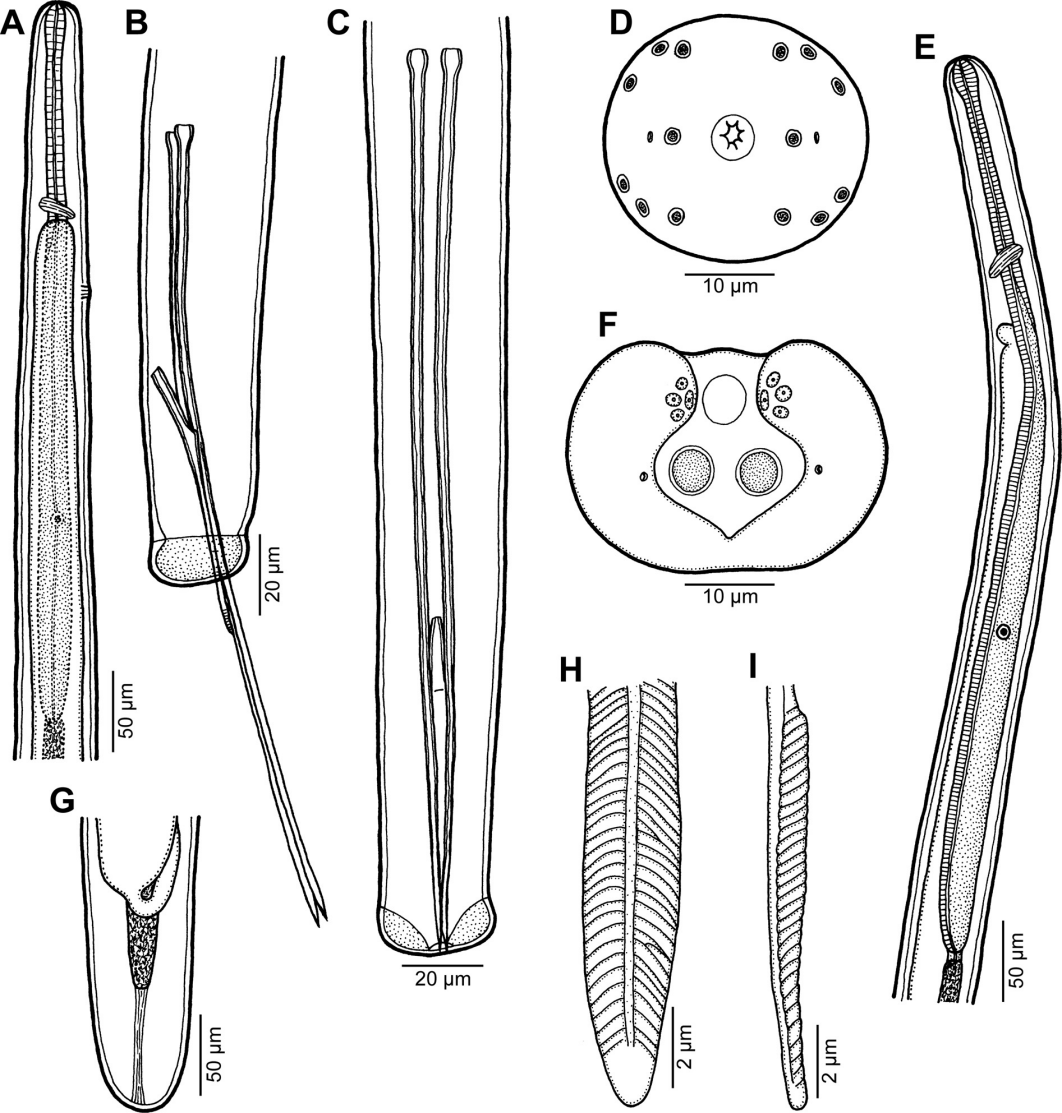
*Philometra jordanoi* (López-Neyra, 1951). A: Anterior end of male, lateral view. B, C: Posterior end of male, lateral and ventral views, respectively. D: Cephalic end of male, apical view. E: Anterior end of mature female, lateral view. F: Caudal end of male, apical view. G: Posterior end of mature female, lateral view. H, I: Posterior end of gubernaculum, dorsal and lateral views, respectively.

**Figure 4.**
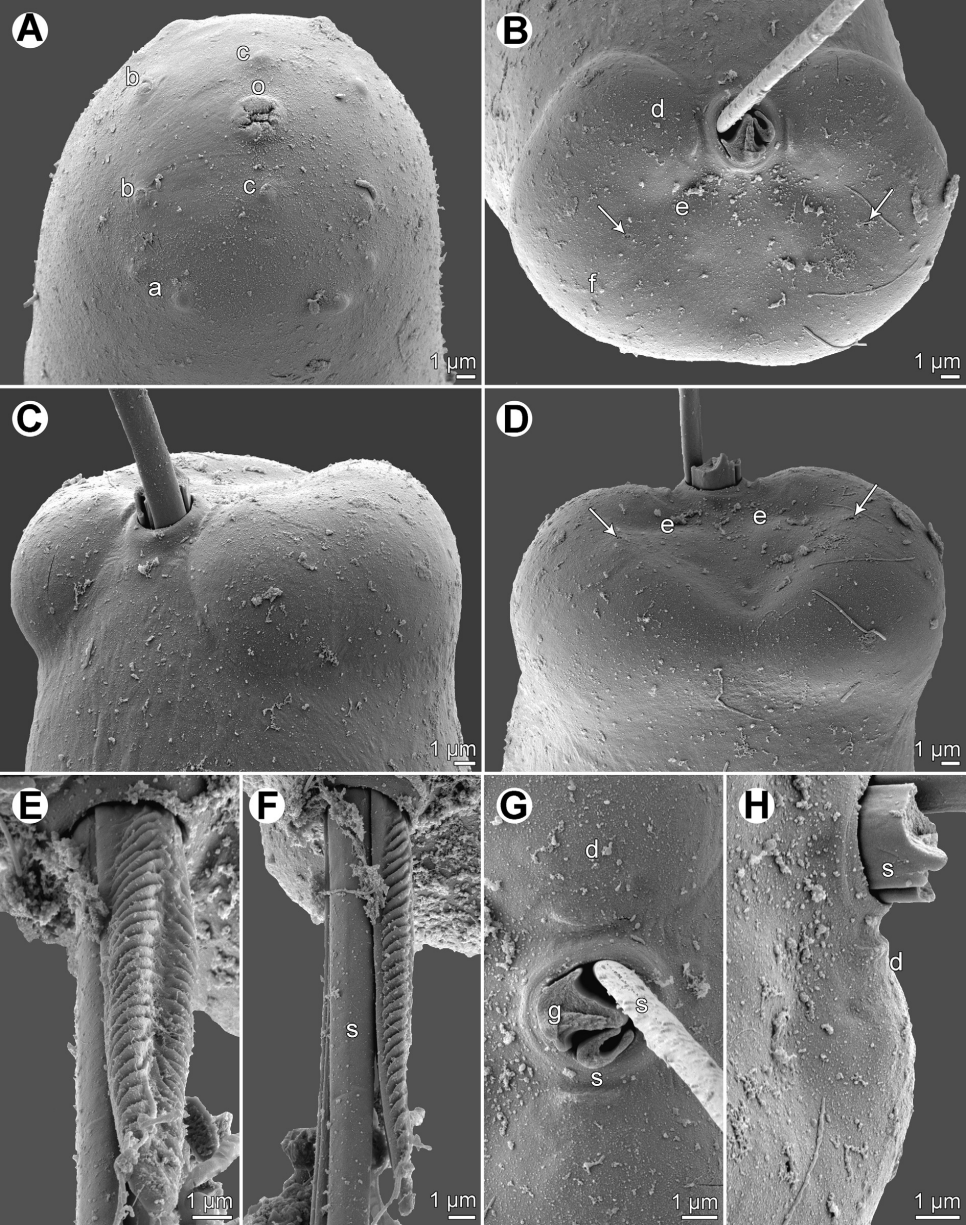
*Philometra jordanoi* (López-Neyra, 1951), scanning electron micrographs of male. A: Cephalic end, subapical view. B: Caudal end, apical view (arrows indicate phasmids). C: Same, sublateral view. D: Same, dorsal view (arrows indicate phasmids). E, F: Distal end of gubernaculum, dorsal and lateral views, respectively. G, H: Region of cloaca, ventral and dorsal views (distal ends of gubernaculum and one spicule broken-away). *Abbreviations*: a, submedian pair of external cephalic papillae; b, submedian cephalic papilla of internal circle; c, lateral cephalic papilla of internal circle; d, group of four adanal caudal papillae; e, large papilla posterior to cloaca; f, caudal mound; g, gubernaculum; o, oral aperture; s, spicule.

Type-host and type-locality: Dusky grouper, *Epinephelus marginatus* (Lowe) (as *E. guaza* (Linnaeus)) (Serranidae, Perciformes), Tétouan (fish market), Morocco. Other localities: see text.

Material from Tunisia.

Host: *Epinephelus marginatus*. After identifying the fish based upon morphological characteristics, identification was confirmed via barcoding. We obtained sequences (GenBank KU739519–KU739521) from three specimens; only one (corresponding sequence: KU739521) had mature gonads and was used in this study. The three sequences were almost identical, with 1–2 bp differences; BLAST results show that our sequences were identical or almost identical (99–100% similarity) to several sequences all identified as *E. marginatus*, such as KC500679 (from Iskenderun Bay, Turkey [7]), JF493449 and JF493450 (from South Africa, unpublished), KM077929 (Senegal [33]) and sequences from Brazil such as KF836469 (unpublished). Our sequences were also identical or almost identical (99–100% similarity) to shorter sequences identified as *E*. *marginatus* such as FN688939 (from France [8]). BOLD gave similar results, but the automatic identification was disrupted by the presence in the database of an “early sequence” labelled as *Epinephelus fasciatus* (Forsskål) (clearly a misidentification). We consider that barcoding definitely demonstrates that our specimens belong to *E*. *marginatus*.

Site of infection: Ovary.

Locality: Sfax (fish market), Tunisia, collected 22 October 2015.

Prevalence and intensity: 1 fish infected/1 fish examined; 7 philometrid specimens.

Voucher specimens: 1 male and 1 female nematode, Muséum National d’Histoire Naturelle, Paris, MNHN HEL555; 1 male and 1 female specimen in the Helminthological Collection of the Institute of Parasitology, Biology Centre of the Czech Academy of Sciences, České Budějovice (Cat. No. N-1110).

#### Description

*Male* (5 specimens): Body whitish, filiform, tapering to both ends, 2.45–2.91 mm long, maximum width at middle 51–60; anterior part of body not narrower just posterior to cephalic end (Fig. 3A). Maximum width/body length ratio 1:48–57; width of cephalic end 24–27, that of posterior end 27. Cuticle smooth. Cephalic end rounded. Oral aperture small, with lobular rim, surrounded by 14 cephalic papillae arranged in 2 circles: external circle formed by 4 submedian pairs of papillae; internal circle formed by 4 submedian and 2 lateral papillae. Small lateral amphids just posterior to lateral papillae of internal circle (Figs. 3D, 4A). Oesophagus 501–564 long, maximum width 18–30, slightly inflated at anterior end; posterior part of muscular oesophagus overlapped by well-developed oesophageal gland with large cell nucleus in middle (Fig. 3A); anterior oesophageal inflation 27–30 long and 18–21 wide. Nerve ring, excretory pore and oesophageal nucleus 144–159, 195–225 and 312–357, respectively, from anterior extremity. Testis reaching anteriorly to level of nerve ring (Fig. 3A). Posterior end of body blunt, with broad, V-shaped mound extending laterally and dorsally (Figs. 3B, C, F, 4B–D). Four pairs of very flat, hardly visible caudal papillae close to each other situated on sides of cloacal aperture on mound (Figs. 3F, 4B–D, G, H). Pair of small phasmids present at about middle of each mound arm (Figs. 3F, 4B, D). Spicules equally long; proximal ends somewhat expanded, distal tips sharply pointed (Fig. 3B, C); length of spicules 213–252, comprising 8% of body length. Gubernaculum 81–84 long, with anterior portion somewhat dorsally bent; length of anterior bent part 24–27, representing 30–32% of entire gubernaculum length; posterior end of gubernaculum with 2 dorsolateral longitudinal parts bearing numerous transverse lamella-like structures demarcating smooth field between them and with two ventral longitudinal grooves (Figs. 3B, C, H, I, 4E–G). Length ratio of gubernaculum and spicules 1:2.61–3.11. Spicules and gubernaculum well sclerotized, yellowish, anterior part of gubernaculum colourless.

*Nongravid female* (2 specimens): Body whitish, filiform, tapering to both ends, 2.48–2.94 mm long, maximum width at middle 48–51. Maximum width/body length ratio 1:52–58; width of cephalic end 24–27, that of posterior end 21. Cuticle smooth. Cephalic end rounded (Fig. 3E), its detailed structure not studied by SEM. Oesophagus provided with large oesophageal gland at its posterior portion, extending from level of nerve ring to end of oesophagus; length of entire oesophagus 600–642 (comprising 22–24% of body length), maximum width including gland 27–30; anterior end of oesophagus slightly inflated at anterior end (Fig. 3E); anterior inflation 27–30 long and 18–21 wide. Nerve ring 147 from anterior extremity; excretory pore indistinct in largest specimen, in smaller specimen situated at 231 from anterior end of body. Small ventriculus 6 long and 9 wide. Intestine narrow, ending blindly, attached by short ligament to body wall near caudal extremity. Vulva absent. Uterus empty. Caudal end rounded (Fig. 3G).

#### Remarks

This species was originally described by López-Neyra [9] as *Sanguinofilaria jordanoi*, based solely on female specimens collected in the ovary of *E*. *marginatus* [syn. *E*. *gigas* (Brünnich)] obtained from the market in Tétouan, Morocco (probably caught in the Mediterranean Sea). Later the species was transferred to *Philometra* as *P*. *jordanoi* [36]. The genus *Sanguinofilaria* Yamaguti, 1941 was subsequently synonymized with *Philometra* [31]. The original description of *P*. *jordanoi* [9] was inadequate. Later Moravec et al. [24], based on available nematode specimens from the ovary of wild *E*. *marginatus* collected in the Mediterranean Sea near the Balearic Islands, Spain and those from wild and cultured *E*. *marginatus* in the Tyrrhenian Sea off Sicily, Italy, provided a somewhat more detailed description of philometrid gravid females, which was, more or less, in agreement with that of *P*. *jordanoi*. Nevertheless, the authors identified this material as *P*. *lateolabracis*, a species described from females collected in three species of perciform fishes off Japan [35], and designated *P*. *jordanoi* to be its junior synonym.

Subsequently, Moravec and Genc [18], based on available body fragments of nematode gravid females from the ovary of *E*. *marginatus* in the Mediterranean Sea off Turkey (Iskenderun Bay), described the female anterior end including cephalic structures of these nematodes and the first-stage larva from the uterus. They again identified these nematodes as *P*. *lateolabracis*. From the same locality (Iskenderun Bay off Turkey) and the same host species, *P*. *lateolabracis* was also reported by Genc et al. [4].

Merella et al. [10] were the first to provide the description of the male (based on a single available specimen studied by LM) of a philometrid collected from the ovary of *E*. *marginatus* in waters near Majorca, Spain, which was identified as *P*. *lateolabracis*. But in their subsequent paper [11], they re-erected *P*. *jordanoi*, to which they assigned the above-mentioned male specimen.

However, Quiazon et al. [30] were the only ones to discover the males of *P*. *lateolabracis* from the type-host in Japan and provided their detailed description based on LM and SEM examinations, which enabled a comparison with other gonad-infecting *Philometra* spp. with described males. Their study showed that *P*. *lateolabracis* is a specific parasite of *Lateolabrax japonicus* (Cuvier) (Lateolabracidae) and that all previous records of this parasite from many other fish species were apparently based on misidentifications. Comparison of the males of *P*. *lateolabracis* and those of *P*. *jordanoi* confirmed the validity of the latter species [12].

It is apparent that all the previous records of *P*. *lateolabracis* in *E*. *marginatus* in the Mediterranean region [4, 18, 24] concerned, in fact, *P*. *jordanoi*. Also, the nematodes designated as *Philometra* sp. from the ovary of *E*. *marginatus* in Iskenderun Bay off Turkey [1] should be assigned to this species.

The present detailed study of the males of *P*. *jordanoi*, including the first use of SEM, made it possible to describe some new taxonomically important morphological features in this species, such as the presence of lamellate structures on the distal end of the gubernaculum, the number and distribution of cephalic and caudal papillae, the character of the male caudal mound, the structure of the male oesophagus, the location of the nerve ring and excretory pore, and the morphology of the mature female. As compared with other gonad-infecting congeneric species parasitizing serranid fishes, *P*. *jordanoi* is remarkable for its rather long spicules, which may attain up to 265 μm [11]. The comparison of *P*. *jordanoi* with other species from the gonads of serranids is more apparent from the following key.

## Key to gonad-infecting species of *Philometra* parasitizing fishes of the family Serranidae

1. Males unknown. Body length of gravid female 40–60 mm. In *Serranus cabrilla* (Linnaeus); Mediterranean Sea region ………………………....................…………….. *P*. *serranellicabrillae*
2. Both males and females known …………………………….........................……………..………….. 2
3. Spicules 432–468 μm long. Gravid female 65–85 mm long. In *Epinephelus morio* (Valenciennes); Gulf of Mexico ……………..............................………………..…. *P*. *margolisi*
4. Spicules shorter than 300 μm ……………………………….........................………………...………. 3
5. Spicules longer than 160 μm ……………………………….........................………………...…..…… 4
6. Spicules shorter than 160 μm ……………………………..........................…………………………… 9
7. Spicules conspicuously distended between second and fourth quarter of their length; spicules 168–186 μm long, length of gubernaculum 120–138 μm. In *Epinephelus bleekeri* (Vaillant); Indian Ocean (Bay of Bengal) ………………………..............…………...… *P*. *tropica*
8. Spicules slender, not markedly distended ……………..................………….……….…………… 5
9. Length of gubernaculum at most 87 μm ……………………...................………………………… 6
10. Gubernaculum longer than 90 μm ………………………………….....................……….…………. 8
11. Pair of large caudal papillae posterior to cloaca absent. Length of spicules 201–219 μm, gubernaculum 78–87 μm long; length ratio of gubernaculum and spicules 1:2.52–2.77. In *Epinephelus costae* (Steindachner); Mediterranean Sea ……....……………... *P*. *tunisiensis*
12. Pair of large caudal papillae posterior to cloaca present ………………….........……………. 7
13. Length of spicules 192–195 μm, gubernaculum 84 μm long; length ratio of gubernaculum and spicules 1:2.32. In *Epinephelus merra* Bloch; Indian Ocean (Bay of Bengal) ……………… …………………………………………………………………………………………………………………….. *P*. *indica*
14. Length of spicules 213–265 μm, gubernaculum 81–84 μm long; length ratio of gubernaculum and spicules 1:2.61–3.15. In *Epinephelus marginatus*; Mediterranean Sea ……………………………………………………………………...............................………....… *P*. *jordanoi*
15. Length of spicules 171–180 μm, representing 4–5% of male body length; gubernaculum 126–144 μm long. Male caudal mound V-shaped. Body length of male 3.67–4.19 mm. In *Epinephelus coioides* (Hamilton); South Pacific (off New Caledonia) and Indian Ocean (Persian Gulf) ………………………………………............................…………..…………... *P*. *piscaria*
16. Length of spicules 183–228 μm, representing 6–8% of male body length; gubernaculum 129–162 μm long. Male caudal mound U-shaped. Body length of male 2.72–3.59 mm. In *Epinephelus cyanopodus* (Richardson); South Pacific (off New Caledonia) ... *P*. *cyanopodi*
17. Gubernaculum with distinct dorsal barb situated postequatorially and conspicuous dorsal protuberance at posterior end in lateral view. Length of spicules 108–123 μm, gubernaculum 96–108 μm long. In *Epinephelus aeneus* (Geoffroy Saint-Hilaire); Mediterranean Sea …………………………………………………………………………………………… *P*. *aenei*
18. Gubernaculum without dorsal barb situated postequatorially and dorsal protuberance at its end ………………………………………………...........................…………………………….……… 10
19. Body length of male 1.63–1.86 mm. Length of spicules 90–120 μm, gubernaculum 57–66 μm long. Body length of gravid female 178–230 mm. In *Epinephelus adscensionis* (Osbeck); Gulf of Mexico ………………...........................…………..…………….. *P*. *mexicana*
20. Body length of male longer than 1.90 mm ……………………..............……………………… 11
21. Spicules 147–156 μm long, length of gubernaculum 69–84 μm. Testis reaching anteriorly only to posterior end of oesophagus. Body length of males 2.75–3.32 mm, of gravid female 387 mm. In *Epinephelus fasciatus* (Forsskål); South Pacific (off New Caledonia) …………………………………………………………................................………………..……. *P*. *fasciati*
22. Spicules 117–165 μm long. Testis reaching anteriorly at least to level of oesophageal nucleus ……………………………………………………………...................................…………..….... 12
23. Caudal mound of male simple, V-shaped, not dorsally divided …………….……...…...… 13
24. Caudal mound of male consisting of two lateral reniform parts widely separated from each other dorsally ………………………………………………………….........................…………… 14
25. Length of spicules 129–147 μm, gubernaculum 96–111 μm long. Testis reaching anteriorly to region of oesophageal nucleus. Body length of male 2.53–2.91 mm. In *Cephalopholis sonnerati* (Valenciennes); South Pacific (off New Caledonia) ……………………………………………………………………………………………………. *P*. *cephalopholidis*
26. Length of spicules 147–165 μm, gubernaculum 63–93 μm long. Testis reaching anteriorly to level of nerve ring. Body length of male 1.97–2.43 mm. In *Mycteroperca rubr*a; Mediterranean Sea ………………………………………….........................……*P*. *inexpectata* n. sp.
27. Body length of male 3.62–4.07 mm, of gravid female 105 mm. Maximum body width/length ratio of gravid female 1:59. Length of spicules 135–138 μm, gubernaculum 84 μm long. Caudal projections in females absent. Larvae in uterus 618–648 μm long. In *Hyporthodus flavolimbatus* (Poey); Gulf of Mexico ………………..........…….. *P*. *hyporthodi*
28. Body length of male 2.01–3.14 mm, of gravid females 178–230 mm. Maximum body width/length ratio of gravid females 1:131–163. Length of spicules 123–141 μm, gubernaculum 54–93 μm long. Gravid and subgravid females with pair of small papilla-like caudal projections. Larvae from uterus 544–648 μm long. In *Mycteroperca phenax*; western Atlantic Ocean (off USA) including Gulf of Mexico ……...…. *P*. *charlestonensis*

## Conflict of interest

The Editor-in-Chief of Parasite is one of the authors of this manuscript. COPE (Committee on Publication Ethics, http://publicationethics.org), to which Parasite adheres, advises special treatment in these cases. In this case, the peer-review process was handled by an Invited Editor, Dominique Vuitton.
